# Monitoring tetracycline through a solid-state nanopore sensor

**DOI:** 10.1038/srep27959

**Published:** 2016-06-16

**Authors:** Yuechuan Zhang, Yanling Chen, Yongqi Fu, Cuifeng Ying, Yanxiao Feng, Qimeng Huang, Chao Wang, De-Sheng Pei, Deqiang Wang

**Affiliations:** 1 School of Physical Electronic, University of Electronic Science and Technology of China, Chengdu, P.R. China; 2 Chongqing Key Lab of Multi-scale Manufacturing Technology, Chongqing Institute of Green and Intelligent Technology, Chinese Academy of Sciences, Chongqing, P.R. China; 3 Research Center for Environment and Health, Chongqing Institute of Green and Intelligent Technology, Chinese Academy of Sciences, Chongqing, P.R. China; 4 Key Laboratory of Weak-Light Nonlinear Photonics, Ministry of Education, School of Physics, Nankai University, Tianjin, China

## Abstract

Antibiotics as emerging environmental contaminants, are widely used in both human and veterinary medicines. A solid-state nanopore sensing method is reported in this article to detect Tetracycline, which is based on Tet-off and Tet-on systems. rtTA (reverse tetracycline-controlled trans-activator) and TRE (Tetracycline Responsive Element) could bind each other under the action of Tetracycline to form one complex. When the complex passes through nanopores with 8 ~ 9 nanometers in diameter, we could detect the concentrations of Tet from 2 ng/mL to 2000 ng/mL. According to the Logistic model, we could define three growth zones of Tetracycline for rtTA and TRE. The slow growth zone is 0–39.5 ng/mL. The rapid growth zone is 39.5−529.7 ng/mL. The saturated zone is > 529.7 ng/mL. Compared to the previous methods, the nanopore sensor could detect and quantify these different kinds of molecule at the single-molecule level.

Tetracycline (Tet) and its analogues are the world’s most widely used antibiotics, affecting both gram-positive and gram-negative bacteria^1^. Tet inhibits protein synthesis by means of binding to the 30S subunit of the bacterial ribosome, preventing tRNA binding to the A-site^1^,^2^. Furthermore, crystal structures of Tet binding to the *Thermus thermophilus* 30S subunit are determined^3^,^4^. Different approaches have been developed for antibiotic residues detection including Enzyme-linked immunesorbent assay (ELISA)^5^, Liquid chromatography (LC)^6^, Liquid chromatography-tandem mass spectrometry (LC-MS/MS)^7^, Surface Plasmon Resonance (SPR)^8^. Those methods have their own advantages and disadvantages. In general, all of these studies depend on expensive, time-consuming techniques and samples preparation, so there remains need for a simple, low-cost, rapid methodology to detect antibiotics residues in environment.

Tet-Off and Tet-On systems are inducible expression models for studies on eukaryote cells and organisms^9^,^10^,^11^. These systems consist of two essential components: a tet-transactivator (tTA) or reverse tet-transactivator (rtTA) protein and a tetracycline-responsive element (TRE) controlling the Tn10 tetracycline-resistance operon of *Escherichia coli* that regulates genes expression^9^. The Tet-Off system for controlling expression of genes of interest in mammalian cells was first developed in 1992^12^. tTA can induce gene expression from TRE promoters in eukaryotic cells, while in the presence of Tet or dox, that inactivates the tTA-TRE interaction and switches gene expression off (Tet-Off system)^12^. Conversely, in another Tet system (Tet-On system), rtTA can only recognize TRE-regulated target gene to promote gene expression with Tet or dox-dependent^13^.

It has been two decades since the beginning from the study of nanopore-based single-molecule sensor, which could sense protein or DNA molecule by a channel connected by two chambers filled with electrolyte solution^14^,^15^. The development of nanopore-sensor experienced two stages: biological nanopore and solid-state nanopore. The solid-state nanopore attracts more attentions because of its tunable size^16^ and reliable stability^17^. Nanopores could be fabricated in Si_3_N_4_ or SiO_2_ membranes by using focus ion beam (FIB)^18^, transmission electron microscope (TEM)^19^ or the dielectric breakdown method^20^. By monitoring the change of the ionic current, the identity of the analytes could be recognized when they are driven through a nanopore by a fixed bias voltage^21^. The nanopore techniques have been used to detect DNA, RNA, and proteins^22^,^23^,^24^,^25^,^26^,^27^. Also recently, many groups have reported the nanopore sensors using graphene as membrane material for bio-molecules detecting^28^,^29^,^30^,^31^. The solid-state nanopore could detect antibiotics through the complexes between antibiotics and RNA fragment^32^.

Here, we report a new nanopore sensing method, which could detect Tet through the mechanism of Tet-Off and Tet-On systems. As shown in Fig. 1, when rtTA, TRE and rtTA-TRE complex with certain concentrations of Tet pass through a solid-state nanopore with appropriate size, it generates different types of blocking ionic current. Therefore, we could identify the presence of tetracycline with solid-state nanopore technique.

## Results

### Electrophoretic Mobility Shift Assay (EMSA)

**Figure 2:**
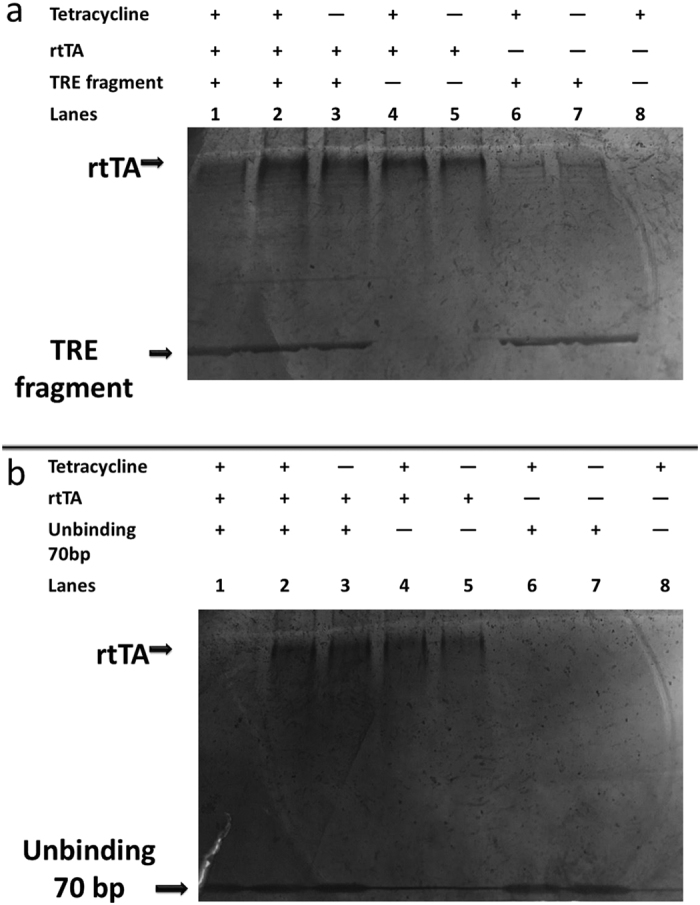
The experimental results of electrophoretic mobility shift assay (EMSA). (**a**) The result of TRE fragments binding with rtTA with or without Tet. (**b**) The result of unbinding 70 bp fragments of non-target as negative control.

In our study, we sought to establish assays to probe target DNA binding with rtTA protein in present of Tet with the Electrophoretic Mobility Shift Assay (EMSA) method. The EMSA experiments were performed to investigate for *in vitro* reconstitution of the rtTA-TRE-Tet complex. The different concentrations of purified rtTA incubated with constant concentrations of TRE fragments without or with excess Tet. rtTA protein was expressed and purified as described in the Methods section. The target DNA of TRE fragment is ~300 bp oligonucleotide duplex containing a minicytomegalovirus (CMV) promoter and TRE full-length sequence that could bind to rtTA with Tet *in vivo*. The 70 bp fragments of the non-target were used as negative control to process the same procedure as TRE fragment. The TRE fragments were amplified by PCR using pTet-On plasmids (Clontech Laboratories, Inc) as template. And we purify them with DNA Gel/PCR Purification Maxiprep kit. In the EMSA assay, the TRE fragment was mixed with a low and a high amount of rtTA (0.5 μM and 2 μM) with Tet (10 μg/ml) complex. Control reactions lacking the miniCMV-TRE, rtTA, Tet or rtTA-Tet complex were also set up, as listed in Table S1. Binding reactions were performed by using a native 10% polyacrylamidegel electrophoresis (PAGE) in a cold room at 4 °C. On the silver stained gels, the lower and upper bands represented the unbound DNA (Fig. 2a,b, lanes 1, 2, 3, 6,7) and complex bound DNA fractions (Fig. 2a, lanes 1, 2, 3), respectively. Silver staining showed that the bands in upper and lower lanes represented rtTA and unbound TRE fragment (Fig. 2a) or non-target (Fig. 2b), respectively. From the EMSA results for complex binding assay, in Fig. 2a, rtTA can bind with parts of TRE fragment (lane 3) without Tet. The lane 5 is rtTA alone. Compared to lane 3, the lane 2 shows that rtTA could interact with more TRE fragment in present of Tet. We also could observe lower concentration of rtTA interacted with less TRE fragment in lane 1, comparing to the higher concentration of rtTA in lane 2. The lane 7 is TRE fragment alone. The lane 8 is Tet alone. The lane 4 includes Tet and rtTA. The lane 6 has Tet and TRE fragment. In Fig. 2b, a random 70 bp oligonucleotides was used as a negative control. TRE fragment was replaced by unbinding oligonucleotides in all reactions, indicating that there is less or no binding ability (lanes 2, 3, 4, 5). All these results above demonstrated that rtTA could bind with TRE fragment in the Tet-On system to activate the transcription of target gene in the present of Tet.

### Detect Tet with solid-state nanopore at different concentrations

**Figure 3:**
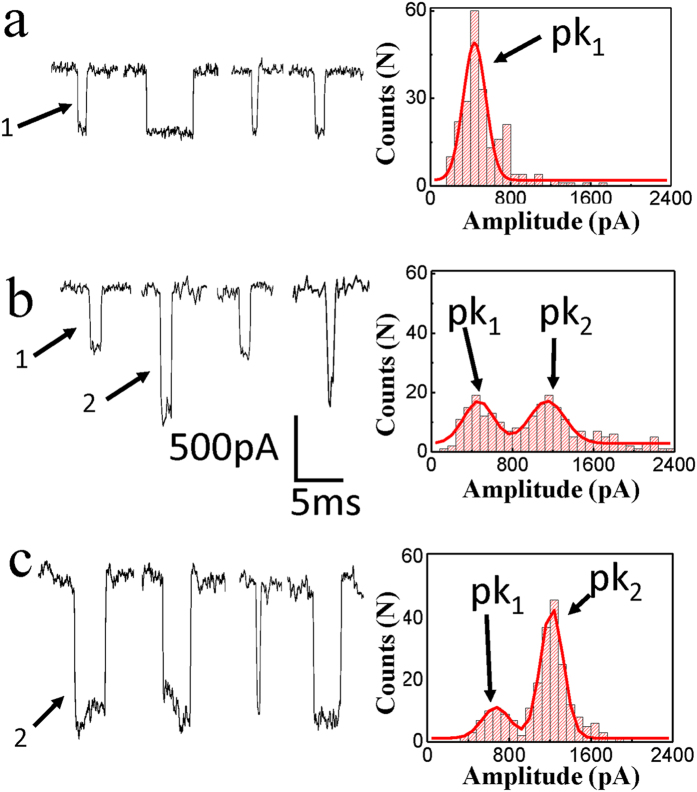
The amplitude histogram distributions and typical events from the mixed rtTA and TRE fragment at different concentrations of Tet. (**a**) 0 ng/mL Tet. (**b**) 50 ng/mL Tet. (**c**) 2000 ng/mL Tet.

In our experiments, The Si_3_N_4_ nanopores around 8 ~ 9 nm in diameter were used to detect rtTA, TRE fragment, the mixed of rtTA and TRE fragment, and their complex respectively. This nanopores in Si_3_N_4_ membrane with 10-nm thickness was taken in this experiment. Each part of nanopore device filled with 1MKCl buffer solution (pH = 8) was applied 200 mV bias voltage. After the ionic current becomes stable, we firstly added 0.5 μL the mixed rtTA and TRE fragment solution in the cis chamber, which contained 200 μL solution. Then, different concentrations of Tet were put into the solution as well. Figure 3 shows the typical current events and histogram distributions with the mixture of rtTA and TRE fragment at different concentrations of Tet ((a) 0 ng/mL Tet. (b) 50 ng/mL Tet. (c) 2000 ng/mL Tet). Our analysis is based on the ionic current parameters, like the duration time (t_dwell_) and the amplitude (I_block_), which was the same method as previous reports^31^,^33^,^34^,^35^. A single combined signal was observed in Fig. 3(a) before adding Tet into the mixed rtTA and TRE buffer solution. After adding Tet to the buffer solution, a new type of current blocked signal has been seen (Fig. 3(b,c)). One peak in Fig. 3(a) and two peaks in Fig. 3(b,c) are observed clearly (pk1 is around 670 pA, while pk_2_ is around 1200 pA). Furthermore, with an increase in the concentration of the added Tet, the height of pk_2_ increased, while that of pk_1_ decreased, clearly suggesting that the changes are ascribed to the complex produced by rtTA, TRE fragment, and Tet. In Fig. S6, the average dwell time also increased as the concentrations of Tet changed from 0 to 2000 ng/mL.

**Figure 4:**
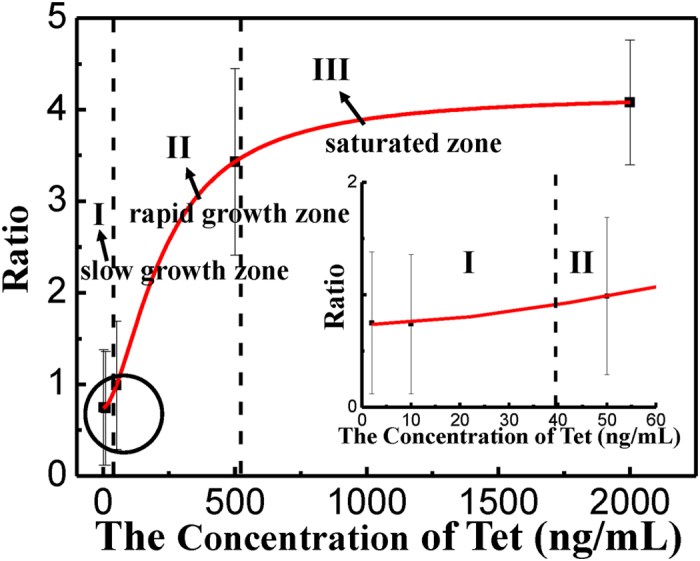
The ratio versus the different concentrations of Tet (2 ng/mL to 2000 ng/mL). The insert shows the enlargement of the black circled area. I is the slow growth zone (0–39.5 ng/mL); II is the rapid growth zone (39.5–529.7 ng/mL); and III is the saturated zone (>529.7 ng/mL).

In order to analyze the recorded current events, we define the ratio as the height of pk_2_ over the height of pk_1_ (pk_2_/pk_1_), which correlates with the concentration of Tet. Figure 4 shows the evolution of the ratio vs. the concentration of Tet. The statistical errors were obtained by calculating the data at three different time period from the same solid-state nanopore In Fig. S8, the overlapping region could bring large errors. In Table S2, it shows the detailed information in Fig. 4. According to the Logistic function, we calculated these three different zones by three special concentrations: EC05 (concentration for 5% of maximal effect, 39.5 ng/ml), and EC80 (concentration for 80% of maximal effect, 529.7 ng/ml). For more details please see Fig. S7. And we used EC05 and EC80 to distinct the three regions. So we could obtain three development zones in Fig. 4, where I is the slow growth zone (0–39.5 ng/mL); II is the rapid growth zone (39.5–529.7 ng/mL); and III is the saturated zone (>529.7 ng/mL). In the slow growth zone, the concentration of Tet is as low as 2 ng/mL, the increase of the ratio is very slow as the concentration of Tet increase. However, when the concentration of Tet exceeds 39.5 ng/mL, the growth of the ratio is very fast, which means this reaction to from the complex may require that the concentration of Tet reaches a certain value. When the concentration of Tet is over than 529.7 ng/mL, the substrates (rtTA and TRE) almost never continue to react. Our experimental results are similar with the values reported from *in vivo* experiments^36^. It is worth noting that our nanopore sensor is very sensitive when the concentration of Tet is between 39.5 ng/mL and 529.7 ng/mL, and even when the concentration of Tet is as low as 2 ng/mL its detection can be provided.

## Discussion

For comparison, Electrophoretic Mobility Shift Assay (EMSA) experiments were performed to investigate the *in vitro* reconstitution of the rtTA-TRE-Tet complex. From the results of EMSA, rtTA interacted with TRE fragment even without Tet, while rtTA could bind more with TRE fragment in present of Tet (see Fig. 2). However, we could not see the Tet concentrations effects from EMSA experiments. It is also not very easy to read the difference before and after adding Tet into the mixed rtTA and TRE solution from EMSA results, but it is evident in the nanopore experimental results. The signals of rtTA were obviously different from that of TRE fragment when the experiments were performed individually (see Figs S2 and 4). There are two peaks for TRE fragment only in Fig. S4(f). It is clear that DNA can be classified into several types when they are driven to get through a nanopore^34^. It does not happen when adding rtTA and TRE fragment in one buffer solution simultaneously. A single combined signal that has higher amplitude was observed in Fig. 3(a) and Fig. S6(b). The weak interaction between rtTA and TRE may just allow TRE to get through the nanopore in one fixed type. However, after adding Tet to the buffer solution, a new type of current blocked signal has been seen (Fig. 3(b,c)).

Our *in vitro* experiments of nanopore sensor for Tet reaction tendency seem similar to the some dox-induced activity *in vivo*, which both have slow growth zone, rapid growth zone and saturated zone^36^. The concentration ranges from 0 ng/mL to 1000 ng/mL. In order to control the use of the tetracycline concentration, the standard for tetracycline in animal products of maximum residue limits (MRL) are not exceeded 100 ng/mL in European Union (EU) and America^37^,^38^. It is worth noting that our nanopore sensor is very sensitive when the concentration of Tet is between 39.5 ng/mL and 529.7 ng/mL, which across the MRL for tetracycline.

## Conclusion

We fabricated nanopores with 8 ~ 9 nm in diameter in Si_3_N_4_ membranes by dielectric breakdown method in 1M pH8 KCl buffer solutions. The nanopore was employed to test rtTA, TRE fragment, the mixture of rtTA and TRE fragment before and after adding Tet. And four different blockade current signals were observed. The most important issue is the significant difference between the two signals that produced by the mixture of rtTA and TRE fragment, and their complex provide a new way to detect Tet on the basis of the solid-state nanopore sensor. To our knowledge, this is the first time to verify Tet using a nanopore sensor based on biological mechanism. In the future, similar techniques may be used to detect other antibiotic substances and become a simple, cost effective, and efficient method to test antibiotics residual in environment.

Moreover, in our experiment, the interaction between protein and DNA under the action of antibiotic was observed clearly. And we analyzed this variation, and deduced the reason of making this change. This result proved nanopore sensor could be used to analyze the basic principle of how protein interacts with DNA. In the future, nanopore technique may help us to understand how viruses infect cells and prevent the disease spreading.

## Methods

### The construction of the recombinant plasmid pET32a-rtTA

pET32a-rtTA plasmid is obtained by means of inserting into HindIII/BamHI-digested pET32a a full-length of rtTA gene cassette, which was amplified by polymerase chain reaction (PCR) from pTet-On plasmid (Clontech Laboratories, Inc) using oligonucleotides rtTA1: (5′cccaagcttatgtctagactggacaagagc′) and rtTA2 (5′cgcggatccttacccggggagcatgtcaag 3′). Recombinant plasmid pET32a-rtTA was prepared according to standard mini-preparation of plasmid method.

### The expression and purification of recombinant protein rtTA

*E. coli* strains directing expression of rtTA were obtained by transforming *E. coli* Rosetta (DE3) with plasmids pET32a-rtTA. Transformed *E. coli* Rosetta DE3 bacteria with plasmids pET32a-rtTA were grown at 37 °C in Luria-Bertani (LB) medium supplemented containing 100 μg/ml ampicillin to an A600 of 0.6–0.8. In order to express recombinant protein rtTA, the culture was then induced with 0.3 mM isopropyl β-D-thiogalactoside (IPTG) and grown continued overnight at 18 °C. Bacteria were harvested by centrifugation (6000 g, 20 min, 4 °C), re-suspended in 50 mM Tris-HCl (pH 7.5), 500-mM sodium chloride, 0.2% Triton-X100, 10% glycerol, 10-mM imidazole, 5 mM 2-mercaptoethanol, and frozen at −80 °C.

The expressed recombinant protein rtTA was purified using following procedure below. Bacteria were thawed and sonicated on ice in the Ni-Native buffer. After centrifugation (9500 rpm, 30 min, 4 °C), the supernatant was collected and solubilized with Ni-Denature-GuHCl. Following centrifugation, cleared lysate was subjected to affinity chromatography using a Ni-NTA Resin (GE Healthcare) and proteins were eluted in Ni-Denature-250 buffer with a linear gradient. The recombinant protein rtTA was finally refolded by a stepwise dialysis and then dissolved in phosphate buffered saline (PBS). Fractions containing histidine (His)-tagged proteins are identified by sodium dodecyl sulphate-polyacrylamide gel electrophoresis (SDS-PAGE) and gel staining with Coomassie Brilliant Blue.

### Nanopore fabrication

The nanopores were fabricated in 10 nm thick Si_3_N_4_ membranes on 200 μm thick silicon with 25 μm × 25 μm opened windows through the dielectric breakdown in solution. These Si_3_N_4_ chips were obtained commercially (*Nanopore solutions, Portugal*).We firstly mounted this type of Si_3_N_4_ chip in fluidic setup. The fluidic setup has two parts, for which both of them has a chamber. The Si_3_N_4_ chips are installed between the two parts of the fluidic cell. Then both of *Cis* and *Trans* chamber are filled with buffer solutions (1.0 M KCl, 10 mMTris-HCl, pH = 8.0). Two Ag/AgCl electrodesare put in each of chambers for containing the solutions. A Keithley 2450 equipment was applied for offering the current pulse through the two electrodes. A Labview program controls Keithley 2450 to apply a current pulse and then measure conductivity of the membrane through an IV curves. So we can monitor the nanopore size in real time through an equation below:


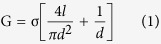


where σ represents the bulk conductivity of the solution; *l* represents the thickness of the membranes; *d* represents the effective diameter of the nanopore. This method allows us to fabricate stable nanopores rapidly. Figure S1 showed a TEM picture of a nanopore fabricated by this method and several IV curves of different sizes nanopores. In our experiment, the nanopores with 8 ~ 9 nm in diameter were used.

## Additional Information

**How to cite this article**: Zhang, Y. *et al*. Monitoring tetracycline through a solid-state nanopore sensor. *Sci. Rep.* **6**, 27959; doi: 10.1038/srep27959 (2016).

## Supplementary Material

Supplementary Information

## Figures and Tables

**Figure 1:**
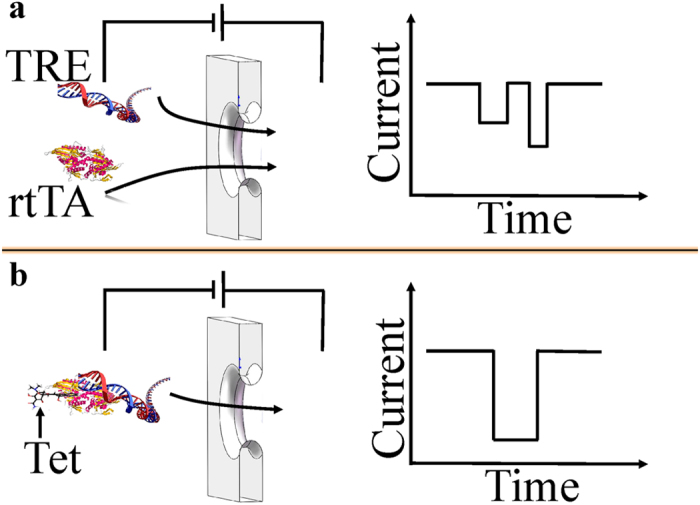
Schematic layout of the experiment: rtTA and TRE fragment combined to each other under the action of Tet to form a complex. (**a**) rtTA and TRE fragment were driven to get through a nanopore. (**b**) The complex was driven to get through a nanopore.
