# Supplementary material for: Monitoring tetracycline through a solid-state nanopore sensor
Source: Sci Rep. 2016 Jun 16;6:27959. doi: 10.1038/srep27959 (PMC4910080; doi:10.1038/srep27959)
Supplement: Supplementary Information [file srep27959-s1.pdf]

Monitoring tetracycline through a solid-state nanopore sensor

Yuechuan Zhang[1,2†],Yanling Chen[3†], Yongqi Fu[1*], Cuifeng Ying[2,4], Yanxiao Feng[2],

Qimeng Huang[2],Chao Wang[3], De-Sheng Pei[3*], Deqiang Wang[2*]

[1]School of Physical Electronic, University of Electronic Science and Technology of China,

Chengdu, P. R. China

[2]Chongqing Key Lab of Multi-scale Manufacturing Technology, Chongqing Institute of Green and Intelligent Technology, Chinese Academy of Sciences,

Chongqing, P.R. China

[3]Research Center for Environment and Health, Chongqing Institute of Green and Intelligent Technology, Chinese Academy of Sciences, Chongqing, P.R. China

[4]Key Laboratory of Weak-Light Nonlinear Photonics, Ministry of Education,

School of Physics, Nankai University, Tianjin, China

Table of contents:

1. **Experimental setup**

2. **Discriminate rtTA and TRE fragment separately**

3. **Before and after adding Tet into the mixed rtTA and TRE solutions**

4. **Figure S1 to Figure S8**

5. **Table S1 and Table S2**

1. Experimental setup

The fluidic cell was made by PMMA (Polymethyl Methacrylate). Each part of the fluidic cell includes a 200 μL chamber, *Cis* chamber and *Trans* chamber. The nanopore device fabricated by dielectric breakdown was mounted in between the two parts of the fluid cell. This nanopore was the only one channel, which connects the *Cis* and *Trans* chambers. An Axopatch 200B (Molecular Devices, CA) was used to apply voltages and detect ionic current through two Ag/AgCl electrodes that were put into each of the chambers respectively. The signal was digitizedby Axon Digidata 1550 with a low-pass 10 KHz filter. Data were acquired and analyzed with the following softwares, pClamp 10.0 (Molecular Devices) and Matlab-based program. The mean values from amplitude and dwell time histograms (over hundreds of events)were obtained with Gaussian and exponential functions. All of this experiment was performed at room temperature. Every Si_3N_4 chip was immersed in piranha solution for 20 minutes before nanopore fabrication. This process was performed toclean the Si_3N_4 chip and prevent the generation of air bubbles. Before nanopore experiments, rtTA and TRE fragment were put in reaction buffer (deionized water). Then 0-2uL Tet was put in it (estimated concentration of rtTA was 25000 ng/mL, estimated concentration TRE was 14000 ng/mL, while that of Tet was between 0 ng/mL and 20000 ng/mL), which the total volume of them is 20 uL, and then let them stand for 60 minutes at room temperature.The Logistic mode fits well with our data. We got three special concentrations of Tet: EC05 (concentration for 5% of maximal effect,39.5 ng/ml), EC20 (concentration for 20% of maximal effect, 99.8 ng/ml),

EC50 (concentration for 50% of maximal effect, 230.0 ng/ml), EC80 (concentration for 80% of maximal effect, 529.7 ng/ml). Three zones were defined depended on these special concentrations (see Fig.3): I is the slow growth zone (0-39.5 ng/mL); II is the rapid growth zone (39.5-529.7 ng/mL); and III is the saturated zone (>529.7 ng/mL).

2. Discriminate rtTA and TRE fragment separately

The Si_3N_4 nanopore around 8.5 nm in diameter with 10-nm thickness was used in this experiment. The resistance of this nanopore was around 25 MΩ. Figure S3 gave the typical signals and events. The blockage current was clearly observed when both biomolecules passed through the nanopore. Two different signals were observed clearly. Hundreds of events were plotted in Fig.S5(a) and (b), where each point represents a single event, and they shows the results of rtTA and TRE fragment. Figure S5(c) and (d) shows the histogram of t_{dwell}. FigureS5 (e) and (f) are the histograms of I_{block}. It is clear that the events were clustered together for all of them from the figures. In order to clearly describe the characteristics of data distribution, we used exponential and Gaussian fittings for the histograms of dwell time and amplitude, respectively. The value of t_1 (about 0.31s) is almost two times larger than that of t_2 (0.15s), whereas t_1 and t_2 are the attenuation factors of exponential fitting results of rtTA and TRE fragment. As can be seen from Fig. S5(e) and (f), there were one peak (pk_1) around 250pA for rtTA and two peaks (pk_{21} and pk_{22}) that one is around 250pA, the other is around 430pA for TRE fragment. It is clear that the events were clustered together for all of them from the figures. We could obviously

discriminate the rtTA and TRE fragment based on the dwell time and amplitude values.

3. Before and after adding Tet into the mixed rtTA and TRE solutions

We tested the mixed rtTA and TRE fragment with nanopore firstly with 8.5 nm nanopore in diameter. After adding Tet to this buffer solution, the signal was changed significantly. All types of this variation trend are showed in Figure S6. There are two different areas showed in Fig. S6(a): the blue area and the red area, which represented the events of putting rtTA and TRE fragment in one buffer solution before and after adding Tet. There are almost no overlaps in these two different areas.To analyze data accurately, exponential and Gaussian modes were adopted to fit the distributions of dwell time and amplitude. From Fig. S6(c) and (d), the value of t_4 (about 3.7s) was much larger than that of t_3 (about 0.3s). Figure S6(b) shows the amplitude distribution of before and after adding Tet: the position of $pk_3$is around 310 pA and the position of $pk_4$is around 475pA. rtTA and TRE fragment combined each other under the action of Tet and produced a much larger complex. This complex could stay too much longer in nanopore and produce a higher blocked current signal. From the results of EMSA, rtTA interacted with TRE fragment even without Tet, while rtTAbinded more with TRE fragment in present of Tet. This result was observed more clearly in our nanopore experiment. The signals of rtTA were obviously different from that of TRE fragment when the experiments were performed individually. There are two peaks for TRE fragment only in Fig. S5(f).It does not happen when rtTA and TRE fragment were added into one buffer solution simultaneously. A single combined signal that has

higher amplitude was observed in Fig. S6(b).The interaction between rtTA and TRE may just allow TRE to get through the nanopore in one fixed type, which may be the reason of producing different signals. After adding Tet into this solution, there is a significant change in both amplitude and dwell time. This reason is clear: rtTA and TRE could combine to each other to form a much bigger complex under the action of Tet, and this complex would stay too much longer time in nanopore and produce much higher amplitude.

FIG.S1

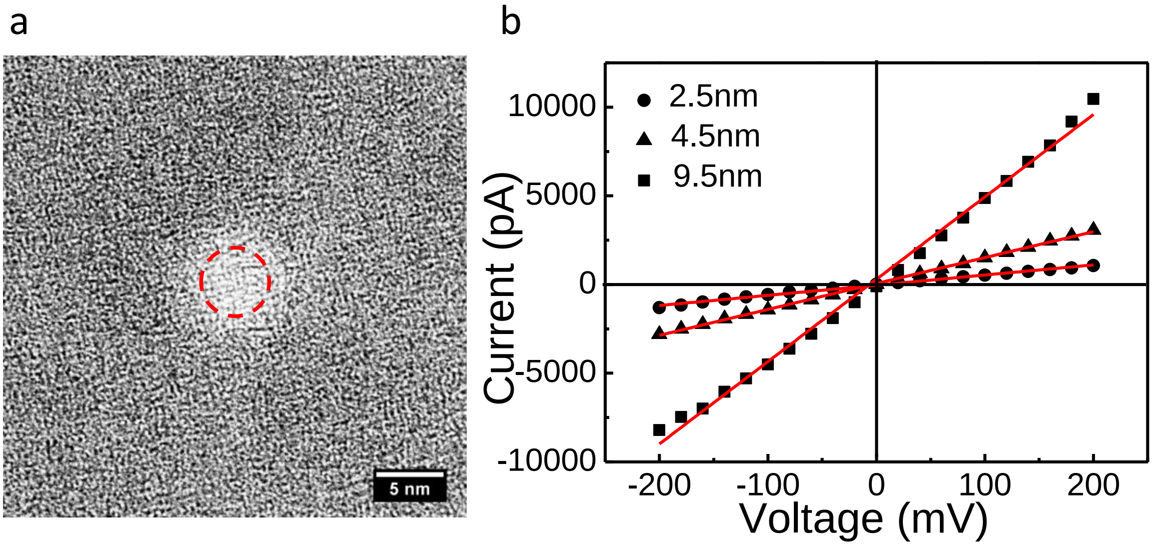

FIG.S1 (a) A picture of a nanoporefabricated by dielectric breakdown. (b)Three IV curves of different sizes nanopores in 10-nm thick Si_3N_4 membranes measured in 1M KCl pH8 buffer solutions, and the top left corner shows their sizes.

FIG.S2

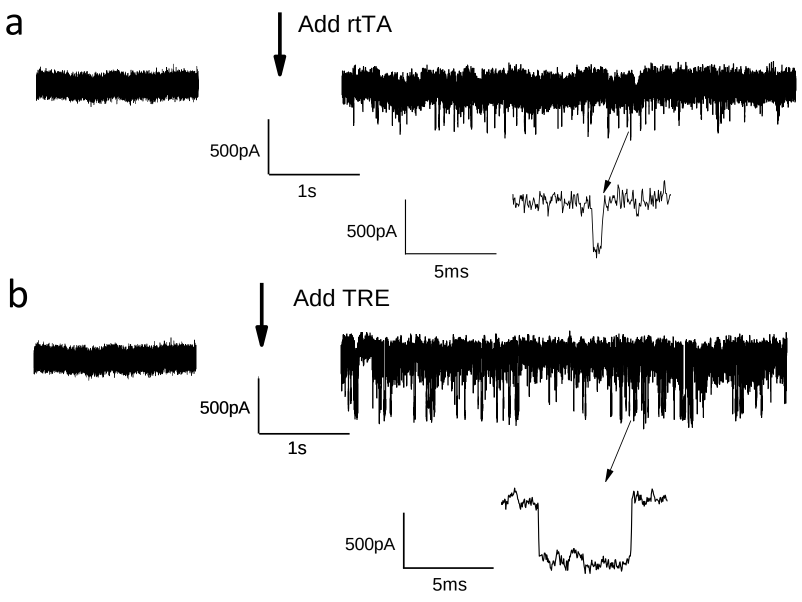

FIG.S2 Ionic current signals and typical events for rtTA and TRE fragment individually. (a) The open-pore ionic current and the ionic current signals for rtTA only; (b) The open-pore ionic current and the ionic current signals for TRE fragment only

FIG.S3

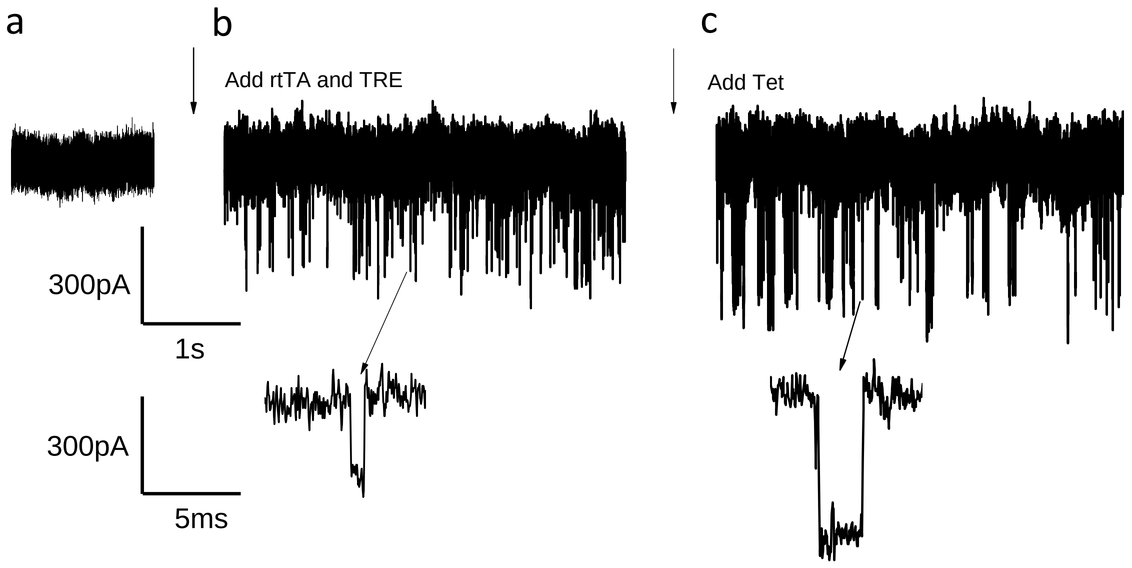

FIG.S3 Ionic current signals for different states before and after adding Tet into the mixed rtTA and TRE fragment solutions. (a) The open-pore ionic current; (b) The ionic current for the mixed rtTA and TRE fragment before adding Tet; (c) The ionic current for the mixed rtTA and TRE fragment after adding Tet.

FIG.S4

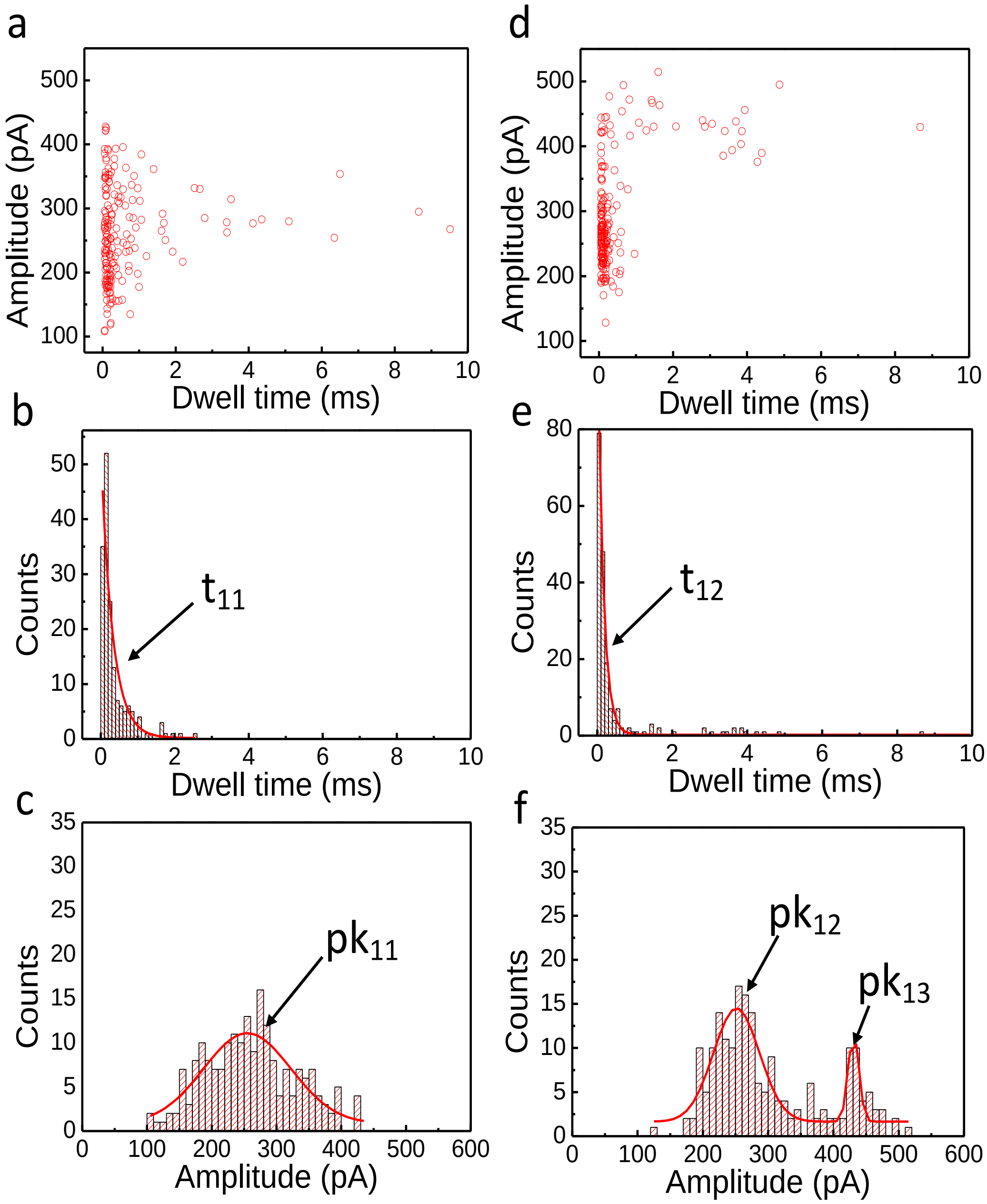

FIG.S4 Experimental results for rtTA and TRE individually. (a) Scatter plot of recorded events at 200 mV bias voltage with rtTA. The points are determined by dwell time and amplitude, and each of them represents a single event. (b) Histogram of dwell time with Exponential fitting for rtTA. (c) Histogram of amplitude with Gaussian fitting for rtTA. (d) Scatter plot of recorded events at 200mV bias voltage with TRE fragment. (e) Histogram of dwell time with exponential fitting for TRE

fragment. (f) Histogram of amplitude with Gaussian fitting for TRE fragment.

FIG.S5

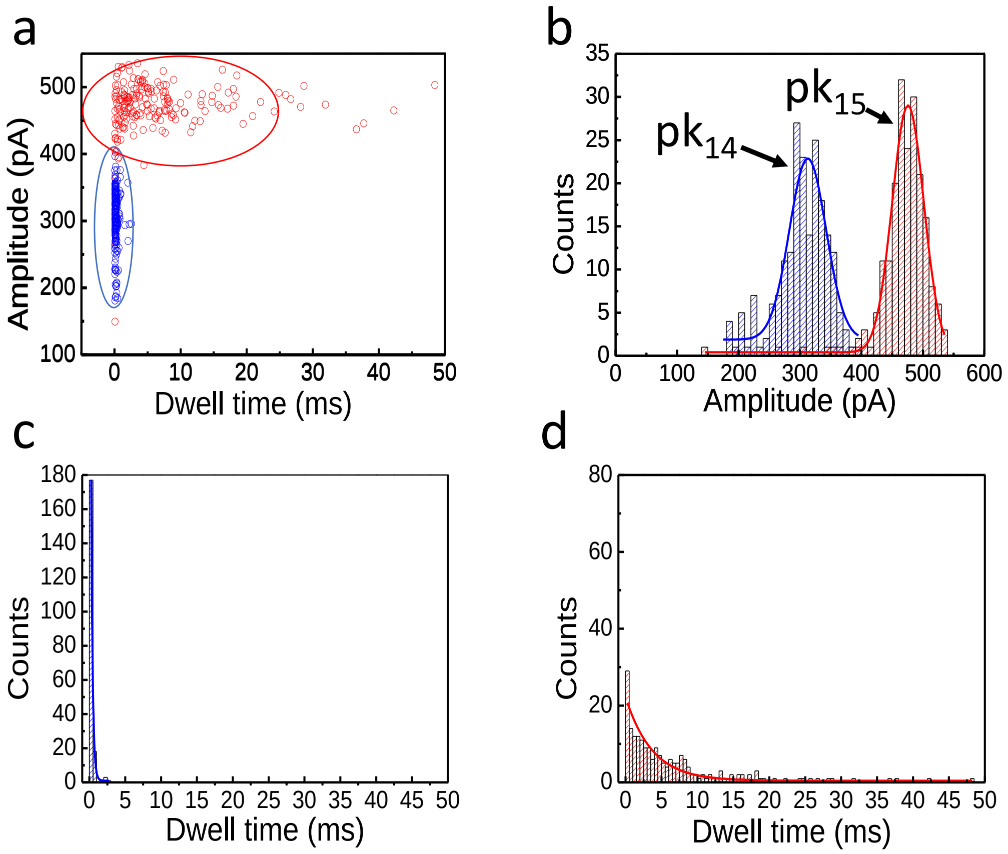

FIG.S5 Experimental results for the mixed rtTA and TRE solutions before and after adding Tet. (a) Scatter plots of recorded events at 200 mV bias voltage. The points are determined by dwell time and amplitude, and each of them represents a single point. The blue points represent the events of mixture of rtTA and TRE fragment before adding Tet, and the red points represent that after adding Tet. (b) Histograms of amplitude for mixture of rtTA and TRE fragment before (blue) and after (red) adding Tet. (c) Histogram of dwell time for mixture of rtTA and TRE fragment before adding Tet. (d) Histogram of dwell time for mixture of rtTA and TRE fragment after adding Tet.

FIG.S6

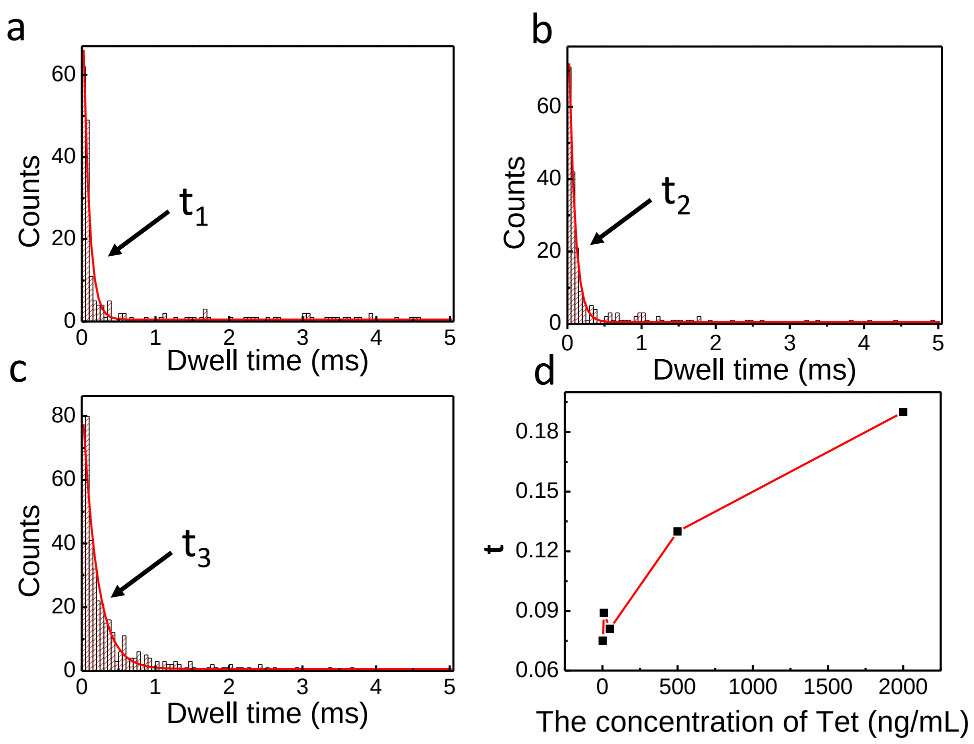

FIG. S6 The average dwell times at different concentrations of Tet. (a) The average dwell time with exponential fitting for 0 ng/mL Tet. (b) The average dwell time with exponential fitting for 50 ng/mL Tet. (c) The average dwell time with exponential fitting for 2000 ng/mL. (d) The average dwell time t vs. the concentration of Tet. Where, t_1 is about 0.079 ms, t_2 is about 0.081 ms, t_3 is about 0.19 ms.The values of them are very close, especially when the concentration of Tet is very low. However, the value of t increases as the concentration of Tet increases, which means high concentration of Tet produce more complex.

FIG.S7

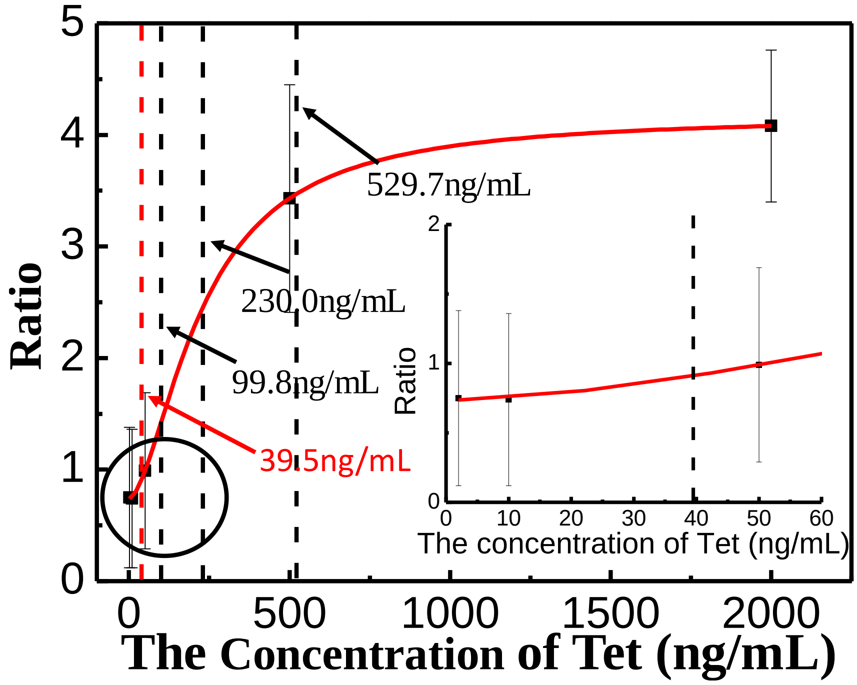

FIG. S7 The ratio versus the different concentrations of Tet (2ng/mL to 2000ng/mL). The insert shows the details of circled areas at the left-bottom corner of this picture. The Logistic mode fits well with our data. We got four special concentrations of Tet: EC05 (concentration for 5% of maximal effect,39.5 ng/ml),EC20 (concentration for 20% of maximal effect, 99.8 ng/ml), EC50 (concentration for 50% of maximal effect, 230.0ng/ml), EC80 (concentration for 80% of maximal effect, 529.7 ng/ml).

FIG.S8

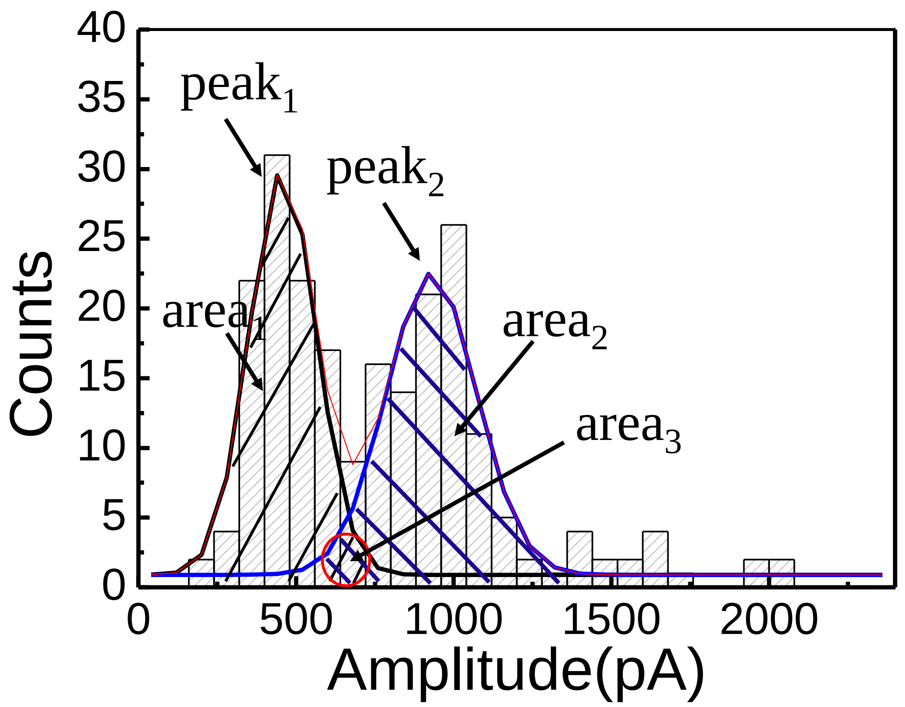

FIG.S8 Histograms of amplitude from the mixed rtTA and TRE fragment at 10 ng/mL Tet. We defined area1 and area2 as the peak areas for peak1 and peak2. The overlapping region (area3) between area1 and area2 could bring large errors when the ratio between peak1 and peak2 is calculated.

Table S1: Experimental conditions for Electrophoretic Mobility Shift Assay (EMSA)

Lanes	rtTA(μl)	TRE fragment/ Unbinding 70bp (μl) (0.6 μM)	Tetracycline(μl) 200μg/ml	Milli-Q water(μl)	Loading buffer (μl)	Total volume(μl)
**1**	5 (2μM)	5	1	0	9	20
**2**	5 (8μM)	5	1	0	9	20
**3**	5 (8μM)	5	0	1	9	20
**4**	5 (8μM)	0	1	5	9	20
**5**	5 (8μM)	0	0	6	9	20
**6**	0	5	1	5	9	20
**7**	0	5	0	6	9	20
**8**	0	0	1	10	9	20

Table S2: Ratio vs. concentration of Tet

Concentration of Tet (ng/mL)	Mean value of the Ratio	Standard Deviation	Total events number
**2**	0.75	0.63	241
**10**	0.74	0.62	220
**50**	0.99	0.7	230
**500**	3.43	1.02	181
**2000**	4.08	0.68	224
